# Methylation pattern of *CDH13* gene in digestive tract cancers

**DOI:** 10.1038/sj.bjc.6602095

**Published:** 2004-08-03

**Authors:** K Hibi, Y Kodera, K Ito, S Akiyama, A Nakao

**Affiliations:** 1 Gastroenterological Surgery, Nagoya University Graduate School of Medicine, 65 Tsurumai-cho, Showa-ku, Nagoya 466-8560, Japan

**Keywords:** *CDH13*, oesophageal cancer, gastric cancer

## Abstract

Recently, the loss of *CDH13* (T-cadherin, H-cadherin) gene expression accompanied by *CDH13* promoter methylation was identified in colon cancers. We examined *CDH13* methylation in oesophageal and gastric carcinomas. Five of 37 oesophageal cancers (14%) and 23 of 66 gastric cancers (35%) demonstrated abnormal methylation of the *CDH13* promoter. Abnormal methylation was frequently found in gastric cancers of patients at all clinical stages just as in E-cadherin, another of the cadherin family, suggesting that these cancers could be methylated at an early stage. These results suggested that *CDH13* might play a variety of roles depending on the tissue type.

Advances in molecular genetics have established that several genetic changes, such as the activation of the K-*ras* oncogene and inactivation of the *p53* tumour suppressor gene, are involved in the pathogenesis of colorectal and other cancers (Bos *et al*, 1987; Vogelstein *et al*, 1988; Baker *et al*, 1989). Recently, a growing number of cancer genes are being recognised that harbour methylation in normally unmethylated promoter CpG islands (Jones and Laird, 1999; Baylin and Herman, 2000). This epigenetic change results in no expression of tumour suppressor gene and plays a key role in an epigenetically mediated loss-of-gene function that is as critical for tumorigenesis as mutations in coding regions. In fact, it has been confirmed that hypermethylation of a normally unmethylated CpG island in the promoter region of *p16* correlates with its loss of transcription in various cancers (Gonzalez-Zulueta *et al*, 1995; Merlo *et al*, 1995; Ueki *et al*, 2000). On the other hand, 70–80% of colorectal cancers with microsatellite instability show aberrant promoter hypermethylation and lack expression of hMLH1 (Herman *et al*, 1998).

Recently, the loss of expression of *CDH13* (T-cadherin, H-cadherin) accompanied by *CDH13* promoter methylation was reported in colon cancers (Toyooka *et al*, 2002). *CDH13* encodes a protein belonging to the cadherin family of cell surface glycoproteins responsible for selective cell recognition and adhesion (Takeichi, 1991). Ubiquitous methylation of *CDH13* in colorectal cancers indicated that it occurs at an early stage in the multistage process of oncogenesis. In this report, all colon cancer cell lines that lacked *CDH13* gene expression demonstrated methylation of CpG sites within the putative *CDH13* promoter. Moreover, *CDH13* methylation was detected in 17 of 35 primary colon cancers, suggesting that *CDH13* is a common target for methylation and epigenetic gene silencing in colon cancer and qualifies as a potential colon cancer suppressor gene.

These results prompted us to examine the *CDH13* status of the whole range of digestive tract cancers. It might be possible that *CDH13* was also inactivated by methylation in other digestive tract cancers and is associated with the tumorigenic pathway. In this study, we first examined the methylation status and gene expression of *CDH13* in digestive tract cancer cell lines using methylation-specific PCR (MSP) and reverse transcription–PCR (RT–PCR), respectively. We then examined *CDH13* methylation in oesophageal and gastric carcinomas. The results obtained were then compared to the clinicopathological features.

## MATERIALS AND METHODS

### Sample collection and DNA preparation

Three colorectal cancer cell lines (SW1083, SW1222, and SW1417), one gastric cancer cell line (MKN1), and one oesophageal squamous cancer cell line (TE1) were kindly provided by the Memorial Sloan-Kettering Cancer Center (New York, NY, USA) or purchased from the American Type Culture Collection (Manassas, VA, USA). Two gastric (NUGC3 and NUGC4) and two oesophageal squamous cancer cell lines (NUEC1 and NUEC2) were established in our laboratory. Primary tumours and corresponding normal tissues were obtained at the Nagoya University Hospital from 37 oesophageal squamous cell cancer and 66 gastric cancer patients who had been diagnosed histologically. These samples were obtained during surgery. All cancer specimens contained more than 70% neoplastic cells. This was confirmed using paraffin-embedded tissues stained by haematoxylin and eosin. Oral or written informed consent, as indicated by the institutional review board, was obtained from all patients. All tissues were quickly frozen in liquid nitrogen and stored at −80°C until analysis. Cell line and tumour DNA were prepared as described previously (Hibi *et al*, 1998).

### Bisulphite modification and MSP

DNA from tumour and normal tissue specimens was subjected to bisulphite treatment as described previously (Hibi *et al*, 2001). The modified DNA was used as a template for MSP. Primer sequences of *CDH13* for amplification were described previously (Sato *et al*, 1998). The primers for the unmethylated reaction were: CDH13UMS (sense), 5′-TTGTGGGGTTGTTTTTTGT, and CDH13UMAS (antisense), 5′-AACTTTTCATTCATACACACA, which amplify a 242 bp product. The primers for the methylated reaction were: CDH13MS (sense), 5′-TCGCGGGGTTCGTTTTTCGC, and CDH13MAS (antisense), 5′-GACGTTTTCATTCATACACGCG, which amplify a 243 bp product. The PCR amplification of modified DNA samples consisted of one cycle of 95°C for 5 min, 33 cycles of 95°C for 30 s, 60°C for 1 min, and 72°C for 1 min for the unmethylated reaction or 29 cycles of 95°C for 30 s, 70°C for 1 min, and 72°C for 1 min for the methylated reaction, and one cycle of 72°C for 5 min. DNAs from TE1 (oesophageal squamous cell cancer cell line) and SW1417 (colon cancer cell line) were used as positive controls of *CDH13* amplification for unmethylated and methylated alleles, respectively. The methylation status of SW1417 cells has already been examined previously (Toyooka *et al*, 2002). Controls without DNA were performed for each set of PCR. In all, 10 *μ*l of each PCR product was directly loaded onto nondenaturing 6% polyacrylamide gels, stained with ethidium bromide, and visualised under UV illumination. Each MSP was repeated at least three times.

### Reverse transcription–PCR (RT–PCR)

First-strand cDNA was generated from RNA as described previously (Hibi *et al*, 1991). The PCR amplification consisted of 30 cycles (95°C for 30 s, 55°C for 1 min, and 72°C for 1 min) after the initial denaturation step (95°C for 2 min). The primers used were: CDH13-S (sense), 5′-TTCAGCAGAAAGTGTTCCATAT, and CDH13-AS (antisense), 5′-GTGCATGGACGAACAGAGT. Primer sequences were described previously (Sato *et al*, 1998). The predicted size of PCR product was 208 bp. The housekeeping gene, *β*-actin, was used as an internal control to confirm the success of the RT reaction.

### Statistical analysis

The *χ*^2^ (Fisher's exact) test and Student's *t*-test were used to examine the association between *CDH13* promoter methylation and clinicopathological features.

## RESULTS

**Figure 1:**
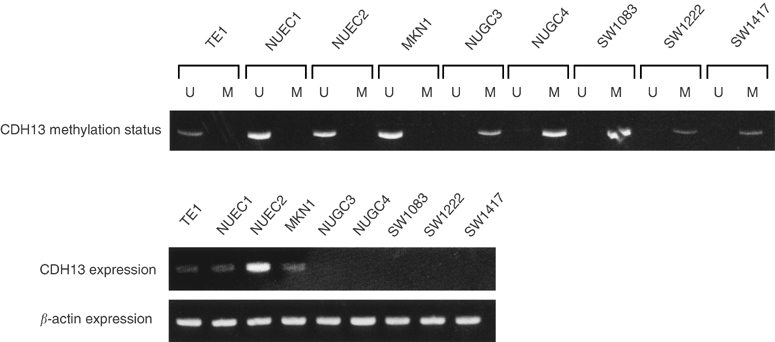
Representative MSP of *CDH13* promoter in digestive tract cancer cell lines. The presence of a visible PCR product in lane U indicates the presence of unmethylated genes; the presence of PCR product in lane M indicates the presence of methylated genes. All colon cancer cell lines (SW1083, SW1222, and SW1417) and two gastric cancer cell lines (NUGC3 and NUGC4) that demonstrated only methylation of the *CDH13* promoter lacked *CDH13* gene expression, while *CDH13* was expressed in other cell lines with unmethylation of the *CDH13* promoter.

We first examined the methylation status of *CDH13* in digestive tract cancer cell lines using MSP. DNA from all three colorectal cancer cell lines (SW1083, SW1222, and SW1417), two of three gastric cancer cell lines (MKN1, NUGC3, and NUGC4), and none of three oesophageal cancer cell lines (TE1, NUEC1, and NUEC2) exhibited abnormal promoter methylation of *CDH13* gene (Figure 1

). To confirm the status of *CDH13* gene according to the methylation pattern, we next examined *CDH13* expression in these cell lines using RT–PCR. Three colon and two gastric cancer cell lines that demonstrated only methylation of the *CDH13* promoter lacked *CDH13* gene expression, while *CDH13* was expressed in all other cell lines with unmethylation of the *CDH13* promoter including one gastric and three oesophageal cancer cell lines (Figure 1).

**Figure 2:**
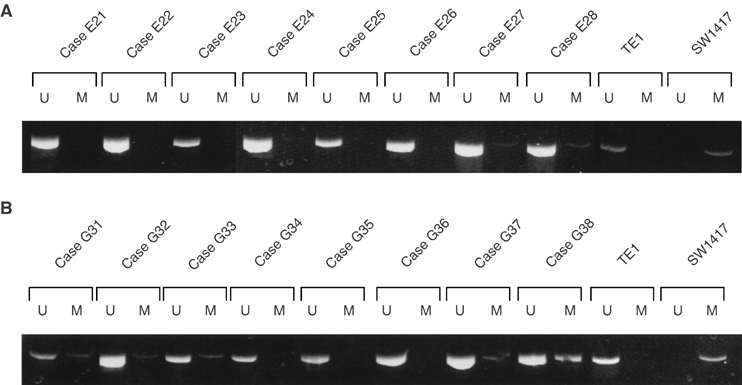
Representative MSP of *CDH13* promoter in primary oesophageal and gastric cancers. In each case, modified DNA from TE1 and SW1417 were used as positive controls for unmethylated and methylated alleles, respectively. (**A**) Primary oesophageal cancers. Cases E27 and E28 exhibited methylation. (**B**) Primary gastric cancers. Cases G31, G32, G33, G37, and G38 exhibited methylation.

Subsequently, we examined whether aberrant methylation could be detected in primary oesophageal and gastric cancers. Five of 37 oesophageal cancers (14%) and 23 of 66 gastric cancers (35%) demonstrated abnormal methylation of the *CDH13* promoter. In our previous study, *CDH13* methylation was detected in 27 of 84 primary colorectal cancers (32%) (data not shown). Representative results of MSP analyses of *CDH13* promoter are shown in Figure 2

. As a control, we screened for *CDH13* methylation in the corresponding normal epithelial DNA of 37 oesophageal and 66 gastric cancer patients. No methylation was found in the normal DNA of this control group. As Figure 2 showed, all cases exhibited unmethylation to a greater or lesser extent. Therefore, it might be possible that the *CDH13* gene expression has not been inhibited completely in these cancers. It might also be possible that DNA derived from inflammatory and interstitial cells among cancer cells exhibited unmethylation because it is impossible to exclude these cells completely from cancer cells obtained for this study.

After methylation analysis of all samples, clinicopathological data were correlated with these results. Sex, age, extent of tumour, clinical stage, lymph node metastasis, histology, and prognosis were not significantly correlated with representations of abnormal methylation in oesophageal or gastric cancers (Tables 1

**Table 1:** Clinicopathological features and *CDH13* promoter methylation in oesophageal cancer

			***CDH13* methylation**		
**Clinicopathological feature**	**Variable**	**No. of cases**	+	−	***P*-value**
Sex	Male	32	5	27	>0.999^a^
	Female	5	0	5	
Age		37	64.8±10.0^b^	62.3±7.8	0.356^c^
Maximal size (cm)		37	5.4±1.3	4.6±1.9	0.528^c^
Extent of tumour	⩽mt^d^	13	0	13	0.140^a^
	mt <	24	5	19	
TNM stage	0–2	9	0	9	0.463^a^
	3, 4	28	5	23	
Lymph node	+	21	3	18	>0.999^a^
Metastasis		16	2	14	
Prognosis	Dead	22	4	18	0.629^a^
	Alive	15	1	14	
Time alive (month)^e^		22	7.8±3.8	19.7±16.3	0.1672
Total		37	5	32	

a Fisher's exact test.

b Mean±s.d.

c Student's *t*-test.

d mt=muscular tunic.

e Dead people only.

and 2

**Table 2:** Clinicopathological features and *CDH13* promoter methylation in gastric cancer

			***CDH13* methylation**		
**Clinicopathological feature**	**Variable**	**No. of cases**	+	−	***P*-value**
Sex	Male	49	17	32	0.964^a^
	Female	17	6	11	
Age		66	62.7±12.5^b^	61.1±9.7	0.571^c^
Extent of tumour	⩽mt^d^	20	6	14	0.586^a^
	mt<	46	17	29	
TNM stage	0–2	35	12	23	0.919^a^
	3, 4	31	11	20	
Lymph node	+	38	14	24	0.692^a^
Metastasis		28	9	19	
Prognosis	Dead	32	11	21	>0.999^a^
	Alive	34	12	22	
Histology	tub^e^	27	10	17	0.756^a^
	por, muc, sig^f^	39	13	26	
Total		66	23	43	

a *χ*^2^ test.

b Mean±s.d.

c Student's *t*-test.

d mt, muscular tunic.

e tub, tubular adenocarcinoma.

f por, poorly-differentiated adenocarcinoma; muc, mucinous adenocarcinoma; sig, signet-cell adenocarcinoma.

). Compared with *CDH13*-unmethylated cancers, *CDH13*-methylated cancers showed a trend towards preferentially invasive (*P*=0.140) and short time alive (*P*=0.167) in oesophageal cancers. On the other hand, abnormal methylation was found in gastric cancers of patients at all clinical stages, suggesting that these cancers could be methylated at an early stage.

## DISCUSSION

Several tumour suppressor genes contain CpG islands in their promoters, prompting many studies investigating the role of methylation in silencing these genes. Many tumour suppressor genes show evidence of methylation silencing, providing a new potential pathway for the deactivation of tumour suppressor genes (Jones and Laird, 1999).

*CDH13*, one among the cadherin family, would be a cell surface glycoprotein responsible for cell adhesion. Recently, it was reported that the promoter of E-cadherin, another of the cadherin family, frequently underwent hypermethylation in human gastric cancers (Tamura *et al*, 2000). Therefore, it is conceivable that *CDH13* was also inactivated in gastric cancers by promoter methylation. In this study, *CDH13* gene was methylated frequently in gastric cancers, suggesting that the inactivation of this gene plays an important role in this cancer while it does not do so in oesophageal squamous cell cancers. Moreover, abnormal methylation was found in gastric cancers of patients at all clinical stages, suggesting that these cancers could be methylated at an early stage. These results suggested that *CDH13* might play various roles depending on the tissue types along the digestive tract.

As previously described, the methylation of *CDH13* gene would not be complete, suggesting that the *CDH13* gene expression has not been completely inhibited in primary cancers. Zheng *et al* (2000) reported previously that a partial methylation pattern was associated with relatively low levels of *p14ARF* in colorectal cancer cell lines. *p14ARF* mRNA was expressed at extremely low levels in fully methylated cell lines, and *p14ARF* expression in the partial methylated LoVo cell line was intermediate. Moreover, partial methylation of *p14ARF* was the most common pattern observed in primary colorectal cancers. Taken together, these findings suggest that the level of *CDH13* gene expression might also be controlled by methylation in primary cancers.

Our results suggested that the aberrant methylation of *CDH13* gene has been shown frequently in oesophageal and gastric cancers. In addition, abnormal methylation was found in gastric cancers of patients at all clinical stages, suggesting that these cancers could be methylated at an early stage. Therefore, *CDH13* methylation could be used as a tumour marker in clinical samples such as serum and stool for the early detection of digestive tract cancers (Hibi *et al*, 2001; Kanyama *et al*, 2003).

Recent studies have shown that it is possible to reverse epigenetic changes and restore gene function to a cell. Treatment with DNA methylation inhibitors can restore the activities of *CDH13* gene and decrease the growth rate of cancer cells. The administration of drugs such as cytosine analogues might soon be able to restore the function of these tumour suppressor genes and slow the rate of cancer progression.
